# Editorial on the “Special Issue in Honor of Dr. Michael Weber’s 70th Birthday: Photodynamic Therapy: Rising Star in Pharmaceutical Applications”

**DOI:** 10.3390/pharmaceutics14091786

**Published:** 2022-08-26

**Authors:** Eduard Preis, Matthias Wojcik, Gerhard Litscher, Udo Bakowsky

**Affiliations:** 1 Department of Pharmaceutics and Biopharmaceutics, University of Marburg, Robert-Koch-Str. 4, 35037 Marburg, Germany; 2 President of ISLA (International Society for Medical Laser Applications), Research Unit of Biomedical Engineering in Anesthesia and Intensive Care Medicine, Research Unit for Complementary and Integrative Laser Medicine, Traditional Chinese Medicine (TCM) Research Center Graz, Department of Anesthesiology and Intensive Care Medicine, Medical University of Graz, Auenbruggerplatz 39, 8036 Graz, Austria

Thousands of years ago, phototherapy or heliotherapy was performed by ancient Egyptians, Greeks, and Romans. However, from the mid-19th century onward, names such as Arnold Rikli, Niels Ryberg Finsen, Downes and Blunt, Oscar Raab, and Hermann von Tappeiner started to appear and paved the way for current experts in the field of photodynamic therapy (PDT), such as Wainwright, Maisch, and Hamblin. Still, only a tiny fraction of PDT’s potential has been realized in the clinical practice guidelines [1].

This Special Issue of *Pharmaceutics* commemorates the tremendous influence Michael Weber’s work had on the use and practical applications of PDT. Looking back after his 70th birthday, in addition to his inventions, patents, and publications, he always found room for hands-on experience as a practicing doctor. He pioneered the clinical use of lasers and photodynamic therapy for nearly 25 years in Germany and several other countries.

Multiple contributions from all over the world emphasize the importance of PDT and signal that there is much more to expect. The mechanism of PDT generally relies on three main components, i.e., light, a photosensitizer (PS), and molecular oxygen; however, it can be subdivided into different applications, which sometimes leads to confusing abbreviations and definitions. When applied against bacteria and fungi, it is often referred to as antimicrobial photodynamic therapy (aPDT) or photodynamic antimicrobial chemotherapy (PACT); against viruses, it is called antiviral photodynamic therapy (aPDT); against cancer cells, most researchers use the unmodified term photodynamic therapy (PDT). To simplify this matter, we divide photodynamic therapy into two parts, i.e., cancer treatment (PDT) or antimicrobial and antiviral therapy (aPDT).

The most extensive section in this Special Issue is aPDT, which comprises five articles and one communication covering bacteria treatment, three articles focusing on wound healing, and one article each looking at antifungal and antiviral therapy. González et al. and Núñez et al. used transition metal complexes, i.e., a homo-bimetallic Re(I) complex [2] and a polypyridine Ir(III) complex [3]. Both proved that these complexes could be effectively used in aPDT. Whereas the first showed an enhanced effect by combining the PS with cefotaxime, the latter successfully used imipenem. Garcia et al. applied Fotoenticine^®^, a new PS derived from chlorin e-6, on a microcosm biofilm [4]. In this ex vivo model, which is closer to the complex in vivo conditions, a qualitative and quantitative reduction in bacterial viability was shown. Regarding biofilms, Battisti et al. highlighted the new fluorescence lifetime imaging, which might offer more insights into the biofilm dynamics and facilitate treatment optimization [5].

Cuadrado et al. and Ayoub et al. used a more technological approach and processed the PS. They incorporated their PS (i.e., zinc menthol-phthalocyanine or parietin) into magnetic nanocomposites [6] or cyclodextrin-inclusion complexes [7].

Additionally, three articles took bacteria treatment one step further and focused more on wound healing. They bridge the gap between in vitro and in vivo conditions [8] and show new in vivo quantification methods [9,10].

Pérez-Laguna et al. assessed the effect of combination therapy against different strains of *Candida* spp. using methylene blue as PS and chlorhexidine [11]. They achieved a reduction in methylene blue concentration while maintaining the same photodynamic efficacy. Another study by Sadraeian et al. compared the effects of UV-C light and aPDT with photodithazine against SARS-CoV-2 pseudovirus [12].

The PDT section consists of five articles covering different aspects. Chai et al. synthesized a new PS that showed beneficial properties (i.e., tracking and ablation) against HepG2 human hepatocellular carcinoma cells [13]. Dobre et al. investigated the gene expression pattern of HT29 cells treated with a new porphyrin derivate that they had previously synthesized and analyzed [14]. Nanosized drug delivery systems are vital when applying the most PS in PDT. Thus, Lehmann et al. and Yeh et al. incorporated their PS in liposomes and lipid-calcium phosphate nanoparticles, respectively [15,16]. While the first group reported the feasibility of liposome nebulization and pulmonary drug delivery, the second successfully treated SCC4 and SAS cells in vitro and in a xenograft model with a combination therapy using EGFR siRNA and PDT. Bartosińska et al. compared three different forms of 5-aminolevulinic acid in treating actinic keratosis and showed that 5-aminolevulinic acid phosphate was superior to the other forms at present due to its higher tolerability and lesser pain [17].

Two articles covering immunomodulatory therapy extended the scope of the two defined sections, PDT and aPDT. Dorst et al. assessed the efficacy of IRDye700DX-loaded liposomes in the treatment of arthritis and provided insights into the difficulties of these treatment regimens [18]. Christensen et al. presented the first-in-human study of 5-aminolevulinic acid against chronic graft-versus-host disease [19]. They used extracorporeal photopheresis combined with photoactivation of the generated protoporphyrin IX and proved the tolerability and safety of the procedure.

Apart from these captivating research articles, seven profound review articles deal with different fields in PDT and aPDT. A broad overview of PDT, from history to future perspectives, was given by Correia et al. [20]. They summarized the essential parameters, discussed advantages and limitations, and emphasized that PDT is a promising therapeutic option. Lange et al. and Ailioaie et al. focused on the use of cyanine-derived dyes and curcumin in PDT, respectively [21,22]. Whereas the first group thoroughly described all used dyes, the second included all technological advances and presented the use of curcumin against different cancer types in detail. Fahmy et al. focused their article on liposomal formulations in PDT, highlighted the versatility of liposomes, and listed an astonishing number of the most recent state-of-the-art studies [23]. The comprehensive review by Piaserico et al. deals with the possible applications of PDT against actinic keratoses. They provided important information on the benefits of post- and pre-treatment strategies that improve the therapeutic efficiency of PDT [24].

A systematic review by Dalvi et al. on using aPDT against periodontitis demanded more robust and well-designed studies due to the substantial flaws limiting their reproducibility [25]. Starting from single PS, along with their different classes and drug-delivery systems, Youf et al. continued to thoroughly describe all possible combinations with aPDT [26].

Overall, photodynamic therapy is a lively topic that can be used in various fields. In 2017, despite some breakthroughs, the world did not seem prepared for PDT and aPDT. We look forward to the changes in clinical practice in the upcoming years.

We thank *Pharmaceutics* (MDPI) and their excellent team for permanently staying by our side and helping us to produce this Special Issue.
